# Executive functions and classroom quality in kindergarten predict peer acceptance in first grade

**DOI:** 10.1038/s41598-025-30409-3

**Published:** 2025-12-04

**Authors:** Arina Shatskaya, Patrik Drid, Alexander Veraksa

**Affiliations:** 1 Laboratory of Childhood Psychology and Digital Socialization, Federal Scientific Center of Psychological and Multidisciplinary Research, Moscow, Russia; 2 Department of Educational Psychology and Pedagogy, Faculty of Psychology, Lomonosov Moscow State University, Moscow, Russia; 3 Faculty of Sport and Physical Education, University of Novi Sad, Novi Sad, Serbia

**Keywords:** CLASS, Peer relationships, School adjustment, Executive functions, Kindergarten, Primary school, Psychology, Human behaviour

## Abstract

The attainment of sufficient peer acceptance profoundly impacts child development and successful school adjustment. This study aims to determine the extent to which child executive functions and classroom quality in kindergarten predict peer acceptance in first-grade schoolers. Data was collected in two different periods, separated by a 1-year interval. At Time 1, classroom quality and executive functions were measured using a sample of 449 children in kindergarten (M = 6.3 years). By the Time 2, the participants were first-grade primary school students and were diagnosed on peer acceptance dimensions: sociometric status, reciprocity, and self-sociometric status (*n* = 203, M = 7.4 years). Regression models were constructed, controlling for sex, age, nonverbal intelligence, child peer acceptance in kindergarten, kindergarten class, and school class as random effects. The results indicated that executive functions in kindergarten are the primary predictor of the sociometric status, as well as the reciprocity. The kindergarten classroom quality, in terms of emotional support, tends to be the most significant predictor of self-sociometric status.

**Supplementary Information:**

The online version contains supplementary material available at 10.1038/s41598-025-30409-3.

## Introduction

The transition to the first grade of primary school constitutes a pivotal and challenging period in a child’s developmental trajectory. A key indicator of successful adaptation for first-graders is the extent of positive acceptance they experience from their peers^1^. Children who are highly regarded by their peers adapt better to school^2^. Positive peer interactions provide a unique context for the practice of social-emotional skills^3^ and personality development^4^, lead to improved school performance^5,6^ and future psychological well-being^7^. Peer acceptance and satisfactory levels of peer communication are associated with increased interest in learning activities^8^. Negative peer relationships contribute to poorer overall mental health in children, leading to increased depressive symptoms and feelings of loneliness^9,10^. Peer rejection is also associated with development of externalizing behavior^11^, low social competence^12^, reduced emotional regulation^13^. In addition, negative peer relationships have a negative impact even on physical health^14^. All these points to the crucial role of positive social relationships with peers in a child’s life and future development.

One of the most robust indicators of peer acceptance is sociometric status. This term refers to a child’s position within a social group, typically classified as accepted or rejected, loved or unloved, and popular or unpopular^15^. It encompasses both emotional factors (liking/disliking, acceptance/rejection) and reputational factors (perceived popularity, visibility, centrality, and social impact)^4,16^. Sociometric status tends to be relatively stable over time, especially rejection, which persists not only within a current group but also when transitioning to a new one^17,18^. Peer relationships, shaped by stable individual differences and preferences, begin forming as early as age three^19^. By entering preschool, children increasingly spend time in fixed peer groups, establishing enduring communication preferences^20^.

One common method for assessing sociometric status involves asking each child to nominate peers with whom they would prefer to participate in joint activities^21^. A greater number of nominations indicates a higher sociometric status. Additionally, two parameters are considered to further elucidate peer acceptance: self-sociometric status (the number of peers a child believes would nominate them) and choice reciprocity (the number of mutual nominations). Together, these metrics provide a nuanced understanding of a child’s sociometric status within a group^21^.

Choice reciprocity represents an essential component of sociometric status assessment. A group member’s position depends not only on the number of received preferences but also on their origin—whether they come from selected peers or from individuals toward whom the person shows indifference^21^. Research findings indicate that subjective satisfaction with one’s group position, significantly influenced by reciprocity, plays a more crucial role in a child’s emotional well-being and school adaptation than objective sociometric status^22,23^. Veraksa et al.^21^ revealed that reciprocity demonstrates a stronger correlation with emotional understanding and inhibitory control (as key factors for peer acceptance) compared to sociometric status itself. Furthermore, first-graders’ self-sociometric status correlates with cognitive control and information processing speed, both of which are critical for successful peer adaptation^24^. Therefore, comprehensive assessment of peer acceptance may extend beyond traditional status measures to incorporate reciprocity and self-sociometric status measures.

Given the established importance of peer acceptance for children’s mental and physical well-being, it is essential to identify the factors that reliably predict this acceptance. The predictors influencing sociometric status remain a subject of ongoing debate^4,21^. Among the most frequently studied individual parameters are aggression^25,26^, level of communication skills^1,27^, and aspects of parental behavior and parenting style^28,29^.

However, most studies focus on emotional and communication domains, while cognitive factors have received less attention^21^. Among key cognitive developments in preschool age, self-regulation is particularly significant. Self-regulation is defined as the ability to organize, plan, and solve problems^30^. Research shows that low self-regulation is generally linked to higher rates of problematic behaviors in childhood, such as antisocial behavior, delinquency, hostility, anger, and aggression^31^. Furthermore, individuals with difficulties in self-regulation, who have limited control over their emotions, behavior, and thoughts, tend to experience fewer satisfying relationships and may face rejection or exclusion within social groups^32^.

In this article, we concentrate on the cognitive component of self-regulation—executive functions (EF)—which are essential for effective self-regulation^33^. Executive functions encompass a set of cognitive processes required for conscious, top-down control of actions, thoughts, and emotions, and are closely tied to neural systems in the prefrontal cortex^34,35^. The main components of EF include working memory (both visual and verbal), which enables the temporary storage and manipulation of information; inhibitory control, which allows one to resist distractions, impulses, and habits in favor of responses suited to the task at hand; and cognitive flexibility, which is the ability to switch between different tasks, rules, or perspectives^36^. Numerous studies have validated this three-component model as effective for analyzing the development of EF in both adults and children^34^. Furthermore, EF reliably predict academic and social success in both school and adulthood, often serving as better indicators than IQ or socioeconomic status, thereby underscoring their importance^37–40^.

There is evidence of a connection between social success in peer relationships and executive function skills, which may serve as an important predictor of sociometric status^41,42^. Poor inhibitory control and working memory negatively impact social interactions, promoting impulsivity and aggression^42^. In both children and adolescents, poor EF is frequently associated with behavioral and disruptive problems^43^, making these children appear less appealing as partners for group activities. Interestingly, while EF have been shown to predict prosocial behavior, some studies suggest they may not strongly influence peer social preferences^41^. These mixed findings underscore the necessity for further research to better understand the role of EF in determining whether children are accepted or rejected by their peers^21,41,43,44^.

In addition to children’s personal traits, peer acceptance can also be affected by their non-individual characteristics of the surroundings. One notable factor is the quality of the kindergarten classroom. This aspect is particularly important given that approximately 90% of Russian children attend kindergarten before entering primary school^45^. To provide context for this study, here is a brief overview of the Russian educational system. The system is structured into several stages: preschool education, general school education, vocational education, and higher education. Of these, only general school education is compulsory, while the others are pursued voluntarily. Generally, preschool education involves attending kindergarten. Russian kindergartens are organized into different classes or groups: the nursery group (available from 1.5 years of age), followed by junior, middle, senior, and preparatory groups. Each group typically lasts one year, after which the child advances to the next group. Upon finishing the final kindergarten group and/or reaching the stipulated minimum age, children transition to the school stage. This stage starts with the first grade and lasts for at least 9 years, with children usually starting first grade between the ages of 6 and 8, averaging at about 7. Both preschool and school education in Russia is free for all citizens, as the vast majority of kindergartens and schools are state-funded^46^. This structure highlights the significance of studying the influence of kindergarten on child development and peer acceptance in countries where kindergarten is a critical component of early education. To evaluate kindergarten’s contribution to this process, this study examined the role of kindergarten classroom quality.

Modern research distinguishes two components of classroom quality: structural and procedural^47^. Structural quality is evaluated using objective indicators, such as class size, teacher–child ratio, availability of materials for play and creative activities, ease of access to these materials, teacher experience, and professional training^48^. Procedural quality, which focuses on interactions within the kindergarten environment, is more challenging to assess. However, it is crucial for understanding relationships between students and adults^49^. These interactions are fundamental to children’s development and learning, as teacher–student relationships and teacher competency have been shown to influence academic achievement from the start of primary school^50,51^.

One of the most used methods for assessing procedural quality is the Classroom Assessment Scoring System (CLASS), which evaluates classroom experience across three domains: Emotional Support, Classroom Organization, and Instructional Support^52^. Each domain comprises several dimensions (ranging from three to four, with a total of 10 dimensions), each assessed on a 7-point scale. Emotional Support measures various aspects of teacher–child interactions, including the ability to create a positive, emotionally receptive atmosphere, responsiveness to the children’s opinions and emotional states, and a flexible, effective response to their challenges. Classroom Organization reflects the teacher’s effectiveness in structuring lessons and managing daily routines, such as efficient task completion and behavior management. Instructional Support evaluates how well the teacher provides opportunities for skill development in children, for example by promoting language skills, conceptual thinking, and offering constructive feedback on the students’ ideas and work.

High-quality teacher–child interactions and effective classroom organization have been identified as protective factors for children’s social development^53^. Children exposed to higher levels of emotional and organizational classroom quality during pre-kindergarten and kindergarten display more advanced social skills and fewer behavioral problems in kindergarten and first grade compared to their peers^54^. Moreover, cumulative classroom quality (encompassing emotional, organizational, and instructional supports) has been shown to explain up to 73% of the variance in children’s problem behavior^55^. Effective classroom organization is associated with better behavioral and cognitive self-control, increased behavioral engagement, and less off-task behavior among children^56^. Emotional support, in particular, exerts a strong influence on the development of inhibition, while instructional support is a significant negative predictor of cognitive flexibility^57,58^. Higher levels of instructional support also correspond with increased empathy and reduced disruptiveness in children^59^. In addition, kindergartens that score highly in overall educational environment quality, especially in terms of emotional support, tend to have children who are more confident in engaging with peers and forming cooperative relationships^60^. Thus, the closeness of the teacher–child relationship is linked to both cognitive and social skills, with the most substantial impact observed in the social domain^61^. These findings suggest that a high-quality classroom environment may be a key predictor of strong social skills and greater peer acceptance in school. However, not all studies have found significant effects of classroom quality on children’s developmental outcomes^62^.

The current study addressed the following research question: What is a more significant predictor of a child’s sociometric status in first grade—a measure of classroom quality during kindergarten or the child’s executive function (EF) level? This period was chosen because it is a crucial time for school adaptation, as children enter a new peer group and form lasting relationships. During this time, emerging relationships with new classmates have meaningful impacts on children’s emotional, personal, and social development, as well as on their physical health^63^. We hypothesize that EF is the most significant predictor of peer acceptance, given that low EF skills are linked to greater impulsivity and more aggressive, disruptive behavior^43,44,64^, which can diminish a child’s social attractiveness. In addition, we propose that among the domains of classroom quality, emotional support is the most important, as it creates a warm, supportive, and respectful environment that fosters children’s autonomy, self-expression^65^, and emotional competence—factors that are likely to enhance a child’s appeal to peers^6,66^.

## Methods

### Participants and procedure

The current study was arranged as a two-wave longitudinal study. Several kindergartens in Moscow were randomly selected. The parents of the children attending these institutions were invited to have their children participate in the diagnostic assessment on a free-of-charge basis. The study’s first wave took place in the fall trimester (Time 1) and involved 449 children (M = 6.3 years, SD = 4.74 months; 48.2% boys), who were recruited from nineteen preparatory classes of Moscow kindergartens. A year later, also in the fall trimester in first grade of primary school (Time 2), 203 of children were retested (M = 7.4 years; SD = 6.3 month; 40% boys). Parents provided written consent for their children’s participation in the research. All children were evaluated and found to have no developmental delays or disabilities. The assessment tasks were administered individually in a quiet environment within the kindergarten or school. Children had the option to withdraw from participation if they felt uncomfortable. Upon completing the assessment, each child received a small, enjoyable sticker as a form of reward.

Data was collected in two different periods, separated by a 1-year interval. At the Time 1 the classroom quality in kindergarten data (emotional support, classroom organization and instructional support), EF skills, non-verbal intelligence, sociometric status were collected. At the Time 2 peer acceptance dimensions (sociometric status, self-sociometric status, reciprocity) were assessed.

### Ethial approval

This study was approved by the Ethics Committee of the Faculty of Psychology, at Lomonosov Moscow State University (the approval no: 2023/18) and conducted in accordance with relevant guidelines and the Declaration of Helsinki. Written informed consent to participate in this study was provided by the participants’ legal guardian/next of kin.

### Measures

#### Peer acceptance

Peer acceptance at Time 2 was evaluated through three measures: the sociometric status of the child, the self-sociometric status of the child, and reciprocity. The peer nomination procedure was adapted from the sociometric experiment developed by Kolominsky^23^. Participants were asked to respond to several questions concerning key aspects of their peer relationships. Question 1: “Which of your classmates would you like to sit at the same desk with?” Question 2: “Which of your classmates do you think would like to sit at the same desk with you?”. This procedure is a reliable measure of peer status^67^.

The sociometric status of a child was assessed by counting the nominations received from peers as a preferred desk neighbor. The self-sociometric status was determined by the number of classmates the child believed would choose them as a desk neighbor. Reciprocity was measured by identifying how many of the child’s chosen peers also selected them as a desk partner. These questions were included because, in the country where the study was conducted, children share a fixed desk partner for an extended period, making comfortable desk sharing important.

At the kindergarten, children were asked a different question: “Which of your classmates would you like to play with?”. Based on their responses to this question, the sociometric status (Time 1) was calculated as a control variable.

#### Executive functions

Children’s EF at Time 1, which includes visual and verbal working memory, inhibitory control, and cognitive flexibility, was assessed utilizing the NEPSY-II neuropsychological test battery^68^. Visual working memory was evaluated using the “Memory for Design” subtest, where children were tasked with recalling the correct arrangement of image components (maximum score: 150). Verbal working memory was assessed through the “Sentence Repetition” subtest, requiring participants to listen to and repeat a series of increasingly complex sentences (maximum score: 34). Inhibitory control was measured with the “Inhibition” subtest, which comprised two tasks: “Naming” in which children rapidly identified various figures, and “Inhibition” where they named the figures in reverse (e.g., stating “square” for “circle”) (maximum score: 20). Cognitive flexibility was assessed using the “Dimensional Change Card Sort” (DCCS), which included three tasks: sorting cards by color, sorting by shape, and sorting according to a specific rule based on card framing (maximum score: 24)^69^. These EF assessment tools were adapted for the Russian children before schooling, proving their reliability^70^.

The overall EF score was calculated by assigning equal weight (25% each) to the four subtests, computing a scaling coefficient for each based on its maximum score, multiplying each raw score by its coefficient, and summing the results to achieve a final score of 100%. The internal consistency of the obtained scale according to the Cronbach’s Alpha coefficient is 0.6, which is an acceptable reliability^71^.

#### Classroom quality

To assess the classroom quality at Time 1, the Classroom Assessment Scoring System (CLASS) methodology was used^52^. This observational tool measures three domains of teacher-child interactions: (a) Emotional Support, (b) Classroom Organization, and (c) Instructional Support. Emotional Support is a composite measure of four dimensions—positive and negative climate, teacher sensitivity, and regard for students’ perspectives. Classroom Organization includes measures of three dimensions—behavior management, productivity, and instructional learning formats. Instructional Support includes three dimensions: such as concept development, quality of feedback and language modeling. All the dimensions are directly scored on a 7-point scale, except for negative climate which is reverse-coded. This method evaluates the quality of work in the classroom, providing an overall CLASS rating for each kindergarten class. Adaptation and assessment of the reliability of the CLASS methodology on a Russian sample was carried out by Bukhalenkova, D.A., Almazova^72^.

#### Control variable

As control variables, we took into account the children’s age and sex at birth, as well as their level of non-verbal intelligence. The inclusion of these control variables is supported by prior findings demonstrating gender differences in peer acceptance skills [e.g., ^73^] and a positive association between intellectual abilities and sociometric status^74^. To assess non-verbal intelligence, Raven’s Colored Progressive Matrices were utilized, as they effectively measure general intellectual abilities^75^. In this test, the child was asked to find the missing fragment of an image in order for it to become complete. The test consists of three series of tasks, each containing 12 images.

#### Data analytical approach

At Time 2, participants were in different school classes, and at Time 1, they were in different kindergarten classes. To account for potential differences between these settings, multilevel regression (generalized mixed modeling) was used with varying random intercepts for school classes and kindergarten classes. Ignoring this nested structure could lead to incorrect assumptions about data independence and misinterpretations of the results. Gelman and Hill suggest that multilevel modeling is appropriate when the intraclass correlation coefficient (ICC) is at least 0.05^76^.

The dependent variables were the dimensions of peer acceptance (sociometric status, self-sociometric status, and reciprocity). The predictors included measures of kindergarten classroom quality (emotional support, classroom organization, and instructional support) and overall executive function (EF). Overall EF was calculated as described in the Executive Functions section, providing a composite measure rather than separate subtests, which minimized model complexity. All Pearson correlations among the subtests were significant (ranging from 0.195 to 0.355). Control variables incorporated into the models were sex at birth, age, non-verbal intelligence, and the child’s kindergarten sociometric status. These controls were included to account for potential continuity in peer acceptance between kindergarten and school^17^.

Thus, the models were specified as follows: peer acceptance dimensions ~ 1 + sex + age + non-verbal intelligence + EF + emotional support + classroom organization + instructional support + kindergarten sociometric status + (1 | school class) + (1 | kindergarten class). All quantitative variables were standardized using z-scores (age, non-verbal intelligence, EF). Models were estimated using the REML method with the bobyqa optimizer. For the 95% confidence intervals, the Wald-CI method and Satterthwaite degrees of freedom were used. A listwise deletion approach was applied for missing data. In addition to multilevel modeling, we employed generalized linear regressions with multi-way cluster-robust standard errors as a robustness check. This conservative approach accounts for potential residual dependence within both school classes and kindergarten class simultaneously. Data for each of the three CLASS scales were grouped into three levels: low, medium, and high CLASS (low—below the 25th percentile; high—above the 75th percentile; medium—from the 25th to the 75th percentile). Data analysis was performed using R (version 4.4.0) with the packages ordinal, lme4, lmerTest, MuMIn, dplyr, sandwich, and multcomp, as well as Jamovi (version 2.6.44).

## Results

### Descriptive statistics

At the first stage of the analysis, descriptive statistics for the study variables were reported (Table 1). For descriptive statistics, the Time 1 sample comprised 449 children from the first wave, while the Time 2 sample consisted of 202 children from the second wave.

**Table 1:** Descriptive statistics for study variables from time 1 and time 2.

Study variables	*N*	Mean	Median	SD	Min	Max	Skewness	Skewness SE	Kurtosis	Kurtosis SE
School peer acceptance (Time 2)										
Sociometric status	203	2.58	2	1.78	0	10	0.62	0.17	− 0.13	0.34
Self-sociometric status	203	1.82	2	1.14	0	3	− 0.47	0.17	− 1.20	0.34
Reciprocity	203	1.02	1	0.957	0	3	0.33	0.17	− 1.16	0.34
Classroom process quality in kindergarten (Time 1)										
Emotional Support	19	5.71	5.57	0.458	4.75	6.44	− 0.43	0.19	− 0.59	0.37
Classroom Organization	19	5.89	6.00	0.597	4.50	6.67	− 0.85	0.19	0.05	0.37
Instructional Support	19	2.55	2.11	1.20	1.00	5.08	0.67	0.19	− 0.40	0.37
Executive functions (Time 1)										
Visual working memory	245	106	113	33.4	17	150	− 0.49	0.16	0.19	0.31
Verbal working memory	244	22.2	22	4.12	14	33	0.47	0.16	0.15	0.31
Inhibitory control	257	12.4	13	2.78	3	17	− 0.14	0.15	− 0.60	0.30
Cognitive flexibility	264	11.5	12	3.37	4	19	− 0.19	0.15	− 0.63	0.23
Non-verbal intelligence (Time 1)	203	15.4	14	8.19	2	34	0.37	0.17	− 0.77	0.34
Sociometric status (Time 1)	229	2.57	2	2.02	0	11	0.93	0.16	1.04	0.32

SD, standard deviation; SE, standard error.

As can be seen from the median values in Table 1, the sociometric status of children in the first grade (Time 2) is similar to that in kindergarten (Time 1). Consequently, the number of choices from peers in primary school is the same as in kindergarten. Additionally, the sociometric status of first-grade children and their self-sociometric status are also equal. This indicates that children generally have a realistic perception of their position among peers. However, the reciprocity, where children believe that the same classmate they selected would also choose them, is lower than both the sociometric and self-sociometric status of the child.

To assess the relationship between sociometric status in kindergarten and school, Spearman correlation analysis was used, since according to the Shapiro–Wilk test, the peer acceptance dimensions demonstrated a non-normal distribution (Shapiro–Wilk W = 0.931, *p* < 0.001 for sociometric status in the first grade, W = 0.818, *p* < 0.001 for self-sociometric status, W = 0.825, *p* < 0.001 for reciprocity, W = 0.889, *p* < 0.001 for sociometric status in kindergarten). Spearman correlation analysis showed that the frequency with which a child was preferred by classmates to play together in kindergarten (sociometric status in kindergarten) was not very strongly, although significantly, correlated with the frequency with which the child was preferred by classmates to sit together at a desk (sociometric status) during first grade (rho = 0.218, *p* = 0.004, *n* = 203).

As for the classroom process quality, the highest classroom quality domain in kindergartens is the classroom organization one. The lowest domain is instructional support.

Attrition from the first wave to the second (from *n* = 449 at Time 1 to *n* = 202 at Time 2) did not lead to any significant systematic differences, either in the sex and age composition of the sample (48.2% boys with an age standard deviation of 5 months at Time 1 versus 40% boys with an age standard deviation of 6 months at Time 2) or in the children’s sociometric status (mean sociometric status of 2.64 at Time 1 versus 2.63 at Time 2; Wilcoxon W = 5203, *p* = 0.973). Nonetheless, age and sex variables were included in the regression models as controls.

### Mixed regression models for peer acceptance dimensions

At the second stage of data analysis, we built generalized mixed models according to the parameters stated in the data analytic approach section (*n* = 203). In the first model, sociometric status (Time 2) was used as the dependent variable. The necessary assumptions were successfully verified (including the test of normality of residuals: Kolmogorov-Smirnov statistics = 0.05, *p* = 0.947; multicollinearity VIF from 1.05 to 2.24; Autocorrelation test Durbin–Watson statistics = 1.79, *p* = 0.280; Levene’s test for homogeneity of residual variances F = 0.81, *p* = 0.370). As a result, the model converged and conditional full model R^2^ = 0.272 (df = 10, LRT X^2^ = 24.5, *p* = 0.006), marginal full model R^2^ = 0.237 (df = 8, LRT X^2^ = 24.01, *p* = 0.002). The evaluation of the fixed effects of the model showed that of all the parameters of the model, only EF (omnibus test F = 9.93, *p* = 0.002) and sociometric status in kindergarten (omnibus test F = 7.5, *p* = 0.006) are significant. The remaining predictors were insignificant (Table 2).

**Table 2:** Fixed coefficients in multilevel regression model of first graders sociometric status.

Effect	β	SE	C.I. Lower	C.I. Upper	z	*p*
(Intercept)	2.827	0.509	1.83	3.82	5.55	< 0.001
Sex	0.37	0.415	− 1.18	0.444	1.14	0.258
Age	0.203	0.179	− 0.5544	0.148	− 1.133	0.257
Non-verbal intelligence	0.416	0.238	− 0.0502	0.883	1.749	0.08
Emotional support	0.426	0.289	− 0.1409	0.993	1.473	0.141
Classroom organization	0.35	0.248	− 0.1361	0.836	1.411	0.158
Instructional support	0.365	0.297	− 0.947	0.218	− 1.226	0.22
EF (Time 1)	0.775	0.246	− 1.2574	− 0.293	− 3.151	0.002
Sociometric status (Time 1)	0.599	0.219	− 1.0276	− 0.171	− 2.742	0.006

SD, standard deviation; SE, standard error.

The conditional R^2^ = 0.272 for the full model explains more than the conditional R^2^ = 0.056 (df = 2, LRT X^2^ = 0.434, *p* = 0.805) for the nested model (sociometric status ~ 1 + (1 | school class) + (1 | kindergarten class) (ΔR^2^ = 0.215, *p* = 0.002). But the evaluation of the random components of the first model showed that for the school class the variation is equal to 0 (SD = 0, ICC = 0.00; LRT = 0.0, df = 1, *p* = 0.983). For the kindergarten class the variation is equal to 0.16 (SD = 0.4, ICC = 0.04; LRT = 0.32, df = 1, *p* = 0.669). Thus, including clustering by kindergarten and school classes results in non-significant random intercept effects. The use of a random effect in the sociometric status model is not very appropriate.

In the second model, self-sociometric status (Time 2) was used as the dependent variable. The assumptions were successfully verified (including the test of normality of residuals: Kolmogorov–Smirnov statistics = 0.109, *p* = 0.225; multicollinearity VIF from 1.05 to 2.24; Autocorrelation test Durbin–Watson statistics = 1.64, *p* = 0.064; Levene’s test for homogeneity of residual variances F = 1.41, *p* = 0.238). As a result, the model converged and conditional full model R^2^ = 0.280 (df = 10, LRT X^2^ = 13.47, *p* = 0.05) and marginal full model R^2^ = 0.094 (df = 8, LRT X^2^ = 8.75, *p* = 0.364). The evaluation of the fixed effects of the model showed that of all the parameters of the model, only emotional support (omnibus test F = 5.16, *p* = 0.023) is significant. The remaining predictors were insignificant (Table 3).

**Table 3:** Fixed coefficients in multilevel regression model of first graders self-sociometric status.

Effect	β	SE	C.I. Lower	C.I. Upper	z	*p*
(Intercept)	2.1145	0.455	1.224	3.0054	4.652	< 0.001
Sex	0.2771	0.465	− 1.188	0.6333	− 0.597	0.551
Age	0.0625	0.151	− 0.359	0.2344	− 0.413	0.68
Non-verbal intelligence	0.111	0.277	− 0.431	0.653	0.402	0.688
Emotional support	0.742	0.327	0.102	1.3821	2.272	0.023
Classroom organization	0.3494	0.299	− 0.935	0.2362	− 1.169	0.242
Instructional support	0.6187	0.331	− 1.267	0.03	− 1.869	0.062
EF (Time 1)	0.2342	0.277	− 0.777	0.309	− 0.845	0.398
Sociometric status (Time 1)	0.0437	0.264	− 0.561	0.4736	− 0.166	0.868

SD, standard deviation; SE, standard error.

The conditional R^2^ for the full model is 0.28, which is higher than the nested conditional R^2^ of 0.167 (df = 2, LRT X^2^ = 4.722, *p* = 0.094) and the full marginal R^2^ of 0.094. The ΔR^2^ in comparison is 0.113 (df = 8, LRT X^2^ = 8.752, *p* = 0.364), indicating a modest improvement when including additional components. However, the evaluation of the random components reveals that for school classes the variance is 0.85 (SD = 0.92, ICC = 0.205; LRT X^2^ = 6.3, df = 1, *p* = 0.012), demonstrating a significant random intercept effect. On the other hand, for the kindergarten classes the variance is 0 (SD = 0, ICC = 0.00; LRT X^2^ = 0.00003, df = 1, *p* = 0.996), indicating a non-significant contribution. Thus, including clustering by school classes and kindergarten classes results in a significant random effect only for school classes, while the random effect for kindergarten classes is not justified. About 20.5% of the variance in self-sociometric status lies between school classes, confirming the necessity of the random intercept.

In the third model, reciprocity (Time 2) was used as the dependent variable. The assumptions were successfully verified (including the test of normality of residuals: Kolmogorov–Smirnov statistics = 0.06, *p* = 0.765; multicollinearity VIF from 1.05 to 2.24; Autocorrelation test Durbin–Watson statistics = 1.5, *p* = 0.05; Levene’s test for homogeneity of residual variances F = 0.59, *p* = 0.442). As a result, the model converged and conditional R^2^ = 0.345 (df = 10, LRT X^2^ = 23.87, *p* = 0.008) and marginal R^2^ = 0.138 (df = 8, LRT X^2^ = 13.24, *p* = 0.104). The evaluation of the fixed effects of the model showed that of all the parameters of the model, only EF (omnibus test F = 5.33, *p* = 0.021) is significant. The remaining predictors were insignificant (Table 4).

**Table 4:** Fixed coefficients in multilevel regression model of first graders reciprocity.

Effect	β	SE	C.I. Lower	C.I. Upper	z	*p*
(Intercept)	3.1594	0.652	0.0118	0.152	− 4.844	< 0.001
Sex	0.0605	0.48	0.3674	2.411	− 0.126	0.9
Age	0.1318	0.265	0.5216	1.473	− 0.498	0.619
Non-verbal intelligence	0.1064	0.308	0.4912	1.646	− 0.345	0.73
Emotional support	0.2939	0.323	0.7129	2.525	0.911	0.362
Classroom organization	0.0579	0.321	0.5027	1.772	− 0.18	0.857
Instructional support	0.24	0.318	0.4219	1.467	− 0.755	0.45
EF (Time 1)	0.6484	0.281	0.3017	0.906	− 2.31	0.021
Sociometric status (Time 1)	0.3365	0.243	0.4436	1.15	− 1.385	0.166

SD, standard deviation; SE, standard error.

The conditional R^2^ for the full model is 0.345, which is higher than the nested conditional R^2^ of 0.255 (df = 2, LRT X^2^ = 10.625, *p* = 0.005) and the full marginal R^2^ of 0.138 (df = 8, LRT X^2^ = 13.244, *p* = 0.104), with a ΔR^2^ of 0.09 (df = 8, LRT X^2^ = 13.244, *p* = 0.104) indicating a modest improvement when additional components are included. The evaluation of the random components reveals that for school classes the variance is 0.715 (SD = 0.846, ICC = 0.1786), while for kindergarten classes it is 0.324 (SD = 0.569, ICC = 0.0896). However, the random effect likelihood ratio tests are not significant for either school classes (LRT X^2^ = 1.992, df = 1, *p* = 0.158) or kindergarten classes (LRT X^2^ = 0.437, df = 1, *p* = 0.509). About 17.9% of the variance in reciprocity lies between school classes, confirming the necessity of the random intercept, while kindergarten classes account for about 9.0%. Thus, in this model, although clustering by school classes and kindergarten classes slightly improves the model fit, the random effects for both clusters are not statistically significant.

In addition to mixed modeling, we employed generalized linear regressions with multi-way cluster-robust standard errors as a robustness check. All model assumptions for sociometric status, self-sociometric status and reciprocity of choices models were satisfactorily met: Levene’s test for homogeneity of residual variances indicated no significant heteroscedasticity (F = 0.598, *p* = 0.442; F = 0.814, *p* = 0.370; F = 0.598, *p* = 0.442 respectively). Shapiro–Wilk tests for normality of residuals confirmed normal distribution of errors (W = 0.980, *p* = 0.204; W = 0,991, *p* = 0.867; W = 0,980, *p* = 0.204 respectively). Residual-predicted scatterplots revealed no concerning patterns in the error distribution. The results, presented in Supplementary Materials Tables, showed general agreement with our main mixed models. For sociometric status (Adjusted R^2^ = 0.221, df = 9, X^2^ = 54.6, *p* = 0.003, AIC = 326, BIC = 350, *n* = 203), kindergarten EF (z = 2.84, *p* = 0.004) and kindergarten sociometric status (z = 2.34, *p* = 0.020) emerged as the most significant predictors. Although p-values increased slightly compared to the mixed models, both variables remained statistically significant, confirming the robustness of these effects (Table S1). For self-sociometric status (Adjusted R^2^ = 0.09, df = 9, X^2^ = 65.7, *p* = 0.03, AIC = 274, BIC = 299, *n* = 203), no significant predictors were found (Table S2). Finally, for reciprocity (Adjusted R^2^ = 0.165, df = 9, X^2^ = 62.1, *p* = 0.005, AIC = 247, BIC = 271, *n* = 203), kindergarten EF constituted the sole significant predictor (z = 4.22, *p* < 0.001) (Table S3).

Thus, for sociometric status and reciprocity of choice, both the mixed model and the generalized linear model with cluster-robust standard errors yielded convergent conclusions regarding significant predictors. However, for self-sociometric status, the generalized linear model with cluster-robust standard errors did not confirm the statistical significance of emotional support that was identified in the corresponding mixed model.

## Discussion

The present longitudinal study aimed to examine how the child EF and classroom quality in kindergarten are related to the first-graders’ peer acceptance. Findings indicated that EF at kindergarten age is significantly predictor of first graders sociometric status and reciprocity. In other words, the degree to which a child selects a classmate as a desk mate, as well as the degree to which that classmate reciprocates the choice, is linked to the EF.

A possible explanation for the results is that EF skills underpin children’s cognitive self-regulation^33^, resulting in behavior that is more structured, effective, and manageable^34^. Working memory directly supports the active representation of self-regulatory goals and strategies drawn from long-term memory^77^. An enhanced working memory facilitates top-down attention control, shielding goal-relevant information from distractions^33^. In contrast, low inhibition control increases susceptibility to impulsive actions and can lead to inadequate social responses^78–80^. Moreover, while working memory and inhibition contribute to a rigid pursuit of goals by preventing distractions, high cognitive flexibility enables children to abandon fewer effective strategies in favor of better ones and to adjust goals as needed^33,81^. Meta-analyses indicate that the combination of these skills results in improved focus and more effective learning outcomes^82^. Conversely, sharing a desk with someone who is frequently distracted, misbehaves, disregards the teacher, or interacts aggressively during group activities can create discomfort and may result in diminished academic performance.

It was shown that sociometric status in kindergarten and first grade correlated significantly, though the relationship was not very strong. This may be partly due to the procedural features of the study: in kindergarten, sociometric status was assessed by asking about a partner for joint play, while in first grade, it was based on the choice of a deskmate for study. Given that play is the primary activity in kindergarten and studying is dominant in primary school^83,84^, this approach reflects the typical activities in each setting. Although sometimes school studies occur with an admixture of play formats, and children also play with each other during breaks. However, according to Sanitary and epidemiological requirements for the organization of education and training, recreation, and rehabilitation of children and youth, first-graders have about 3–5 lessons that last 35–40 min during the school day, and breaks last 15–25 min^85^. In addition, the traditional Russian curriculum requires that children sit at desks—often in pairs—which not only determines spatial arrangements but also structures their interactions, as teachers frequently organize teamwork among deskmates. Thus, assessing sociometric status in terms of play for kindergarteners and educational interaction for first-graders is most appropriate.

Secondly, the modest association between sociometric statuses in kindergarten and first grade may be explained by content differences. Although previous studies indicate that sociometric status tends to be maintained during group transitions^17,18^ and that its stability has moderate to high correlations (around 0.50)^17,86,87^, the length of the interval is typically the main explanatory factor. Notably, procedural features of nomination were ruled out, emphasizing time as the primary factor^17,88,89^. However, Jiang and Cilessen^17^ noted that even after controlling for measurement interval, age, and gender, significant variance remained unexplained. Our results suggest that the new social context in first grade—a setting where previous social dispositions undergo qualitative change^90^—may be even more critical. Vygotsky, in his cultural-historical approach, defined the social situation of development as “the objective place of the child in the system of social relations and the corresponding expectations and demands placed on him by society; the peculiarities of the child’s understanding of the social position he occupies and his relationships with the people around him”. It is completely unique, specific for a given age, exceptional, unique and unrepeatable relationships between a child and the reality surrounding him, primarily social^78^. The educational environment and the form of interaction with adults is one of the most important factors that determine the social situation of a child’s development^91^. While kindergarten emphasizes play-based interactions and has its own set of requirements, the school environment imposes significantly different academic demands, creating distinct social situations. Entering school with new classmates and environmental characteristics introduces new challenges that may influence a child’s choice of companion for academic activities^21^. Consequently, the continuity between kindergarten and first-grade sociometric statuses may be low, as observed in the current study. It is therefore crucial to consider the qualitative differences in these contexts rather than solely the time interval or procedural specifics.

Regarding predictors of self-sociometric status, high classroom quality in kindergarten, particularly in terms of emotional support, is a significant predictor of elevated self-sociometric status among first graders according mixed models. In classrooms with robust emotional support, teachers foster a positive, accepting atmosphere that promotes respectful communication and mutual assistance, thereby enhancing children’s emotional competence and self-evaluation^6^. Emotional competence is central to social adjustment^65,92,93^, and children who experience greater emotional support often develop higher self-confidence and self-esteem^94^, which in turn positively shapes their perception of social acceptance.

However, generalized linear models with cluster-robust standard errors accounting for clustering of data across kindergarten and school classes did not confirm the significance of emotional support for self-sociometric status. Previous studies have also reached similar results. For example, teacher sensitivity as measured by CLASS is not directly associated with peer rejection^95^. Several research on emotional support scales has indicated that negative teacher-student interactions significantly influence peers’ perceptions of teacher disfavor, while positive interactions show no clear effect^96^. Moreover, one study found that, after controlling for gender and problem behavior, teacher feedback accounted for only an additional 3% (negative) and 11–12% (positive) of the variance in peer acceptance^97^. Additionally, teacher feedback on inappropriate behavior predicted peer acceptance during classroom activities at the end of the school year but not during recess^98^. Hence, further research into the relationship between classroom quality and peer acceptance is encouraged^97^.

The ideal analytical approach for the present study design would be to employ a multilevel model, simultaneously incorporating individual- and group-level data. This would allow for the appropriate representation of CLASS variables at the group level, thereby mitigating the risks of pseudo replication and the misestimation of statistical effects. However, at Time 2, only one child remained from kindergarten group 2 and none from group 4. Clark^99^ established that reliable multilevel analysis requires adequate group sizes and sufficient number of groups. At the extremes of data sparseness (two observations per group), the group level variance components are overestimated in the two-level models^99^. Besides, researchers have often expressed concern using multilevel modeling when the number of clusters or groups is low (e.g., groups < 20)^100^. The present data had 19 kindergarten classes. Thus, the use of multilevel modelling is often limited, forcing researchers to use ordinary linear models^99^, as was done in the current study in the first version of the models. In the second version, we attempted to further strengthen the analysis by using cluster-robust standard errors to refine the results. For future studies, we would recommend using multilevel models whenever possible.

A further recommendation is that future research should investigate how peer perceptions vary across different social and instructional contexts, given that the daily experiences of first-graders are characterized by a diversity of interactions, among which play constitutes a significant component. We hypothesize that first graders tend to choose the same peers for both learning and play, given their limited experience with activities that require varied partner qualities. Ivanova^24^ found that, at this age, sociometric status is not yet related to a partner’s academic performance, potentially precluding differentiation of preferences based on academic success—a pattern that appears in older children^101^. Moreover, the factors influencing being chosen as a desk mate versus a play partner likely begin to diverge even in first grade. In the present study, EF significantly contributed to first graders’ sociometric status when selecting desk mates, whereas Ivanova^24^ observed no such association for play partners. This suggests that children as early as first grade may differentiate the criteria by which they select their peers according to the goals of their joint activity. Different bases for peer selection in various contexts may have a compensatory, beneficial effect on maintaining a positive self-image; for instance, a child less favored as a play partner due to a calm, composed nature might be preferred for academic projects for the same reasons—a hypothesis that warrants further investigation.

Thus, EF skills, as the cognitive foundation for self-regulation, emerged as more significant predictors of sociometric status and its reciprocity, representing more “objective” measures of peer acceptance (as assessed from an external perspective). In contrast, the quality of emotional support in the classroom proved to be a more significant predictor of self-sociometric status, serving as a more subjective measure of peer acceptance (based on the child’s self-perception).

### Limitations and future research

Certain limitations weaken the findings of the present research. Firstly, the two-wave study with a 1-year interval presents a limitation, as the extended time frame does not allow for the monitoring of fluctuations in sociometric status and hinders the ability to draw conclusions about the nature of its changes during the transition from kindergarten to school. Second, peer acceptance was assessed solely through the peer nomination procedure, without analyzing the reasons behind children’s choices of classmates. Conducting mini-interviews on this topic could provide valuable insights into the relationship between peer acceptance and various indicators, including EF and classroom quality in kindergarten.

## Conclusions

This longitudinal study underscores the crucial role of child EF skills and kindergarten classroom quality in influencing peer acceptance among first graders. The findings reveal that EF skills serve as significant predictors of sociometric status and its reciprocity, suggesting that children with better EF skills are more likely to be embraced by their peers. Thus, cognitive skill development in preschool years significantly predicts peer social acceptance in elementary school. Conversely, the quality of kindergarten emotional support tends to predict self-sociometric status, highlighting the importance of a nurturing and supportive educational environment in shaping children’s self-perception and social acceptance. Yet, this relationship is negated with cluster-robust errors, emphasizing that analytical choices are paramount for specific samples.

## Supplementary Information

Below is the link to the electronic supplementary material.

Supplementary Material 1

## Data Availability

The datasets generated during and/or analysed during the current study are available from the corresponding author on reasonable request.

## References

[1] Nakamichi, K., Nakamichi, N. & Nakazawa, J. Preschool social-emotional competencies predict school adjustment in Grade 1. *Early Child Dev. Care*. **191**(2), 159–172. 10.1080/03004430.2019.1608978 (2021).

[2] Johnson, C., Ironsmith, M., Snow, C. W. & Poteat, G. M. Peer acceptance and social adjustment in preschool and kindergarten. *Early Child. Educ. J.* **27**, 207–212. 10.1023/B:ECEJ.0000003356.30481.7a (2000).

[3] Laible, D. Attachment with parents and peers in late adolescence: links with emotional competence and social behavior. *Pers. Individ. Differ*. **43** (5), 1185–1197. 10.1016/j.paid.2007.03.010 (2007).

[4] Ilmarinen, V. J., Vainikainen, M. P., Verkasalo, M. & Lönnqvist, J. E. Peer sociometric status and personality development from middle childhood to preadolescence. *EJP* **33** (5), 606–626. 10.1002/per.2219 (2019).

[5] Ladd, G. W., Ettekal, I. & Kochenderfer-Ladd, B. Peer victimization trajectories from kindergarten through high school: differential pathways for children’s school engagement and achievement? *J. Educ. Psychol.* **109** (6), 826–841. 10.1037/edu0000177 (2017).

[6] Morris, C. A., Denham, S. A., Bassett, H. H. & Curby, T. W. Relations among teachers’ emotion socialization beliefs and practices and preschoolers’ emotional competence. *EE&D* **24** (7), 979–999. 10.1080/10409289.2013.825186 (2013). 10.1080/10409289.2013.825186 PMC3804260 24159256

[7] Hartup, W. W. The company they keep: friendships and their developmental significance. *Child. Dev.* **67** (1), 1–13. 10.2307/1131681 (1996). 8605821

[8] Holmes, C. J., Kim-Spoon, J. & Deater-Deckard, K. Linking executive function and peer problems from early childhood through middle adolescence. *J. Abnorm. Child. Psychol.* **44**, 31–42 (2016). 26096194 10.1007/s10802-015-0044-5 PMC4689661

[9] Husky, M. M. et al. Bullying involvement and self-reported mental health in elementary school children across Europe. *Child. Abuse Negl.* **107**, 104601. 10.1016/j.chiabu.2020.104601 (2020). 32570185 10.1016/j.chiabu.2020.104601

[10] Schwartz-Mette, R. A., Shankman, J., Dueweke, A. R., Borowski, S. & Rose, A. J. Relations of friendship experiences with depressive symptoms and loneliness in childhood and adolescence: A meta-analytic review. *Psychol. Bull.* **146** (8), 664–700. 10.1037/bul0000239 (2020). 32406698 10.1037/bul0000239

[11] Menting, B., van Lier, P. A. C. & Koot, H. M. Language skills, peer rejection, and the development of externalizing behavior from kindergarten to fourth grade. *JCPP* **52** (1), 72–79. 10.1111/j.14697610.2010.02279.x (2011). 21143229 10.1111/j.1469-7610.2010.02279.x

[12] Öneren Şendil, Ç. An investigation of social competence and behavioral problems of 5–6 year-old children through peer preference, temperament and gender. Master’s thesis, Middle East Technical University (2010).

[13] Gülay Ogelman, H. & Fetihi, L. Examination of the relationship between emotional regulation strategies of 5-year-old children and their peer relationships. *ECDC* **191** (1), 49–57. 10.1080/03004430.2019.1600513 (2019).

[14] Leigh-Hunt, N. et al. An overview of systematic reviews on the public health consequences of social isolation and loneliness. *Public. Health*. **152**, 157–171. https://doi.org/10.1016/j. puhe.2017.07.035 (2017). 28915435 10.1016/j.puhe.2017.07.035

[15] Cillessen, A. H. N. Sociometric methods. In *Handbook of Peer Interactions, Relationships, and Groups* (eds Rubin, K. H., Bukowski, W. M. & Laursen, B.) (Guilford Press, 2009).

[16] Cillessen, A. H. N. & Bukowski, W. M. Sociometric perspectives. In *Handbook of Peer Interactions, Relationships, and Groups*, 2nd edn (eds Bukowski, W. M., Laursen, B. & Rubin, K. H.) 64–83 (Guilford, 2018).

[17] Jiang, X. L. & Cilessen, A. H. N. Stability of continuous measures of sociometric status: a meta-analysis. *Dev. Rev.* **25** (1), 1–25. 10.1016/j.dr.2004.08.008 (2005).

[18] Fournier, M. A. Adolescent hierarchy formation and the social competition theory of depression. *J. Soc. Clin Psychol*. **28**, 1144–1172. 10.1521/jscp.2009.28.9.1144 (2009).

[19] Parker, J. G., Rubin, K. H., Erath, S. A., Wojslawowicz, J. C. & Buskirk, A. A. Peer relationships, child development, and adjustment: A developmental psychopathology perspective. In *Developmental Psychopathology* (eds. Cicchetti, D. & Cohen, D.J.). 10.1002/9780470939383.ch12 (2015).

[20] Kondratiuk, M. I. The concept of a peer of older preschoolers with different sociometric status. *Preschool­Educ.­Today* **18** (4), 48–56. 10.24412/2782-4519-2024-4124-48-56 (2024).

[21] Veraksa, A. N., Oshchepkova, E. S., Aslanova, M. S. & Yakupova, V. A. Factors of a child’s social development in the senior preschool age. *Vestnik Saint Petersburg Univ. Psychol.* **12** (4), 410–430. 10.21638/spbu16.2022.402 (2022).

[22] Valetova, A. I. The relationship between sociometric status of adolescents and their conflict behavior strategies [Bachelor’s thesis, Belgorod State National Research University]. Open Access Electronic Archive. http://dspace.bsuedu.ru/bitstream/123456789/23511/1/Valetova_Svyaz_16.pdf (2016).

[23] Kolominsky, Y. L. Psychology of the children’s collective. *Narodnaya Asveta* (1984).

[24] Ivanova, M. K. Cognitive and emotional development of first-graders in the Sakha Republic (Yakutia). Vestnik of North-Eastern federal university. *Pedagogics Psychol. Philos.* **4** (32), 101–110. 10.25587/2587-5604-2023-4-101-110 (2023).

[25] da Silva, B. M., Veiga, G., Rieffe, C., Endedijk, H. M. & Güroğlu, B. Do my reactions outweigh my actions? The relation between reactive and proactive aggression with peer acceptance in preschoolers. *Children* **10** (9), 1532 (2023). 37761493 10.3390/children10091532 PMC10528464

[26] Gifford-Smith, M. E. & Brownell, C. A. Childhood peer relationships: social acceptance, friendships, and peer networks. *J. Sch. Psychol.* **41**, 235–284. 10.1016/S0022-4405(03)00048-7 (2003).

[27] van der Wilt, F., van der Veen, C., van Kruistum, C. & van Oers, B. Why do children become rejected by their peers? A review of studies into the relationship between oral communicative competence and sociometric status in childhood. *Educ. Psychol. Rev.* **31**, 699–724. 10.1007/s10648-019-09479-z (2019).

[28] Franz, D. & Gross, A. Child sociometric status and parent behaviors: an observational study. *Behav. Modif.* **25**, 3–20. 10.1177/0145445501251001 (2001). 11151484 10.1177/0145445501251001

[29] Putallaz, M. Maternal behavior and children’s sociometric status. *Child. Dev.* **58**, 324–340. 10.2307/1130510 (1987).

[30] Smirnova, E. O. Spetsifika Sovremennogo doshkol’nogo Detstva [The specifics of modern preschool childhood]. *National’nyi Psikhologicheskii Zhurnal*. **2** (34), 33–40. 10.11621/npj.2019.0205 (2019).

[31] Robson, D. A., Allen, M. S. & Howard, S. J. Self-regulation in childhood as a predictor of future outcomes: A meta-analytic review. *Psychol. Bull.* **146** (4), 324. 10.1037/bul0000227 (2020). 31904248 10.1037/bul0000227

[32] Hladik, J., Hrbackova, K. & Petr Safrankova, A. Peer interaction in class: exploring students’ self-regulation in relation to peer acceptance and rejection. *Cogent. Educ.* **11** (1). 10.1080/2331186X.2024.2343520 (2024).

[33] Hofmann, W., Schmeichel, B. J. & Baddeley, A. D. Executive functions and self-regulation. *TiCS* **16** (3), 174–180. 10.1016/j.tics.2012.01.006 (2012). 10.1016/j.tics.2012.01.006 22336729

[34] Diamond, A. & Lee, K. Interventions shown to aid executive function development in children 4 to 12 years old. *Science* **333** (6045), 959–964. 10.1126/science.1204529 (2011). 21852486 10.1126/science.1204529 PMC3159917

[35] Lerner, R. M., Liben, L. S. & Mueller, U. *Handbook of Child Psychology and Developmental Science, Cognitive Processes* (Wiley, 2015).

[36] Miyake, A. et al. The unity and diversity of executive functions and their contributions to complex frontal lobe tasks: A latent variable analysis. *Cogn. Psychol.* **41** (1), 49–100 (2000). 10945922 10.1006/cogp.1999.0734

[37] Best, J. R., Miller, P. H. & Naglieri, J. A. Relations between executive function and academic achievement from ages 5 to 17 in a large, representative National sample. *Learn. Individ. Differ.* **21** (4), 327–336. 10.1016/j.lindif.2011.01.007 (2011). 21845021 10.1016/j.lindif.2011.01.007 PMC3155246

[38] Stankevich, O. A. Assessment of the skills of first-grade students to carry out self-regulation. *Preschool­Educ.­Today* **18** (4), 57–69. 10.24412/2782-4519-2024-4124-57-69 (2024).

[39] Rzhanova, I. E., Alekseeva, O. S., Britova, V. S. & Burdukova, Y. A. Fluid intelligence in children with learning disabilities. *Psychol. Russ*. **18** (1), 20–34. 10.11621/pir.2025.0102 (2025). 40822636 10.11621/pir.2025.0102 PMC12352357

[40] Moffitt, T. E. et al. A gradient of childhood self-control predicts health, wealth, and public safety. *PNAS* **108** (7), 2693–2698. 10.1073/pnas.1010076108 (2011). 21262822 10.1073/pnas.1010076108 PMC3041102

[41] Zorza, J. P., Marino, J. & Mesas, A. A. Executive functions as predictors of school performance and social relationships: primary and secondary school students. *Span. J. Psychol.* **19**, E23. 10.1017/sjp.2016.23 (2016). 27169746 10.1017/sjp.2016.23

[42] Ellis, M. L., Weiss, B. & Lochman, J. E. Executive functions in children: associations with aggressive behavior and appraisal processing. *J. Abnorm. Child. Psychol.* **37** (7), 945–956. 10.1007/s10802-009-9321-5 (2009). 19408113 10.1007/s10802-009-9321-5 PMC3978175

[43] Jacobson, L. A., Williford, A. P. & Pianta, R. C. The role of executive function in children’s competent adjustment to middle school. *Child. Neuropsychol*. **17**, 255–280. 10.1080/09297049.2010.535654 (2011). 21246422 10.1080/09297049.2010.535654 PMC4075458

[44] Stormshak, E. A. et al. The relation between behavior problems and peer preference in different classroom contexts. *Child. Dev.* **70** (1), 169–182. 10.1111/1467-8624.00013 (1999). 10191521 10.1111/1467-8624.00013 PMC2761650

[45] Kardanova, E. et al. Patterns of First-Graders’ development at the start of schooling: cluster approach based on the results of iPIPS project. *Voprosy obrazovaniya/Educ. Stud. Mosc.* **1**, 8–37. 10.17323/1814-9545-2018-1-8-37 (2018).

[46] Revina, V. E. Analiz mekhanizma gosudarstvenno-chastnogo partnerstva na primere detskih sadov / V. E. Revina, I. D. Koval’chuk // 4-ya Mezhdunarodnaya nauchno-prakticheskaya konferenciya molodyh uchyonyh i specialistov po ustojchivomu razvitiyu, investiciyam i finansovym riskam Finatlon forum: Materialy konferencii, Moskva, 16 aprelya 2024 goda. – Moskva: Moskovskij politekh, 586–597 (2024).

[47] Camilli, G., Vargas, S., Ryan, S. & Barnett, W. S. Meta-analysis of the efects of early education interventions on cognitive and social development. *Teachers Coll. Record*. **112** (3), 579–620. 10.1177/01614681101120030 (2010).

[48] Harms, T., Clifford, R. M. & Cryer, D. *Early Childhood Environment Rating Scale* (Teachers College, 2014).

[49] Brunsek, A. et al. The relationship between the early childhood environment rating scale and its revised form and child outcomes: A systematic review and meta- analysis. *PLoS One*. **12** (6), e0178512. 10.1371/journal.pone.0178512 (2017). 28586399 10.1371/journal.pone.0178512 PMC5461062

[50] Hamre, B. K. & Pianta, R. C. Early teacher–child relationships and the trajectory of children’s school outcomes through eighth grade. *Child. Dev.* **72** (2), 625–638 (2001). 11333089 10.1111/1467-8624.00301

[51] Hamre, B. K. & Pianta, R. C. Learning opportunities in preschool and early elementary classrooms. In *School Readiness & the Transition To Kindergarten in the Era of Accountability*, 49–84 (Brookes, 2007).

[52] Pianta, R. C., Paro, L., Hamre, B. K. & K. M., & *Classroom Assessment Scoring System™: Manual K-3* (Paul H Brookes Publishing, 2008).

[53] Rimm-Kaufman, S. E. & Chiu, Y. J. I. Promoting social and academic competence in the classroom: an intervention study examining the contribution of the responsive classroom approach. *Psychol. Sch.* **44** (4), 397–413. 10.1002/pits.20231 (2007).

[54] Broekhuizen, M. L., Mokrova, I. L., Burchinal, M. R., Garrett-Peters, P. T. & Family Life Project Key Investigators. Classroom quality at pre-kindergarten and kindergarten and children’s social skills and behavior problems. *Early Child. Res. Q.* **36**, 212–222. 10.1016/j.ecresq.2016.01.005 (2016). 26949286 10.1016/j.ecresq.2016.01.005 PMC4774198

[55] Elka, Z. Z. & Zhao, W. Problem behaviors in the early childhood O-Class: investigating Emotional, Organizational, and instructional supports. *Early Child. Educ. J.* 1–12. 10.1007/s10643-023-01507-6 (2023).

[56] Rimm-Kaufman, S. E., Curby, T. W., Grimm, K. J., Nathanson, L. & Brock, L. L. The contribution of children’s self-regulation and classroom quality to children’s adaptive behaviors in the kindergarten classroom. *Dev. Psychol.* **45** (4), 958–972. 10.1037/a0015861 (2009). 19586173 10.1037/a0015861

[57] Bukhalenkova, D., Veraksa, A. & Chursina, A. The effect of kindergarten classroom interaction quality on executive function development among 5-to 7-year-old children. *Educ. Sci.* **12** (5), 320. 10.3390/educsci12050320 (2022).

[58] Veraksa, A., Bukhalenkova, D. & Almazova, O. Executive functions and quality of classroom interactions in kindergarten among 5–6-year-old children. *Front. Psychol.* **11**, 603776 (2020). 33329274 10.3389/fpsyg.2020.603776 PMC7714336

[59] Siekkinen, M. et al. Social competence among 6-year-old children and classroom instructional support and teacher stress. *EE&D* **24** (6), 877–897. 10.1080/10409289.2013.745183 (2013).

[60] Gavrilova, M. N., Veraksa, A. N. & Bukhalenkova, D. A. Connection between the classroom quality of a pre-school institution and children’s outcomes: A theoretic overview. *Tomsk State Univ. J.* **433**, 135–145. 10.17223/15617793/433/19 (2018).

[61] Peisner-Feinberg, E. S. et al. The relation of preschool child-care quality to children’s cognitive and social development trajectories through second grade. *Child. Dev.* **72** (5), 1534–1553 (2001). 11699686 10.1111/1467-8624.00364

[62] Mohamed, A. & Marzouk, S. The association between preschool classroom quality and children’s social-emotional problems. *Early Child. Dev. Care*. **186**, 1–14. 10.1080/03004430.2015.1092140 (2015).

[63] Rimm-Kaufman, S. E. & Pianta, R. C. An ecological perspective on the transition to kindergarten: A theoretical framework to guide empirical research. *JADP* **21** (5), 491–511 (2000).

[64] Farley, J. P. & Kim-Spoon, J. The development of adolescent self-regulation: reviewing the role of parent, peer, friend, and romantic relationships. *J. Adolesc.* **37**, 433–440. 10.1016/j.adolescence.2014.03.009 (2014). 24793391 10.1016/j.adolescence.2014.03.009 PMC4021015

[65] Bukhalenkova, D., Veraksa, A., Aslanova, M., Williams, P. & Larsson, J. Sociometric status and child development in preschool age. In *Child Development in Russia: Perspectives from an International Longitudinal Study*, 223–238 (Springer International Publishing, 2022).

[66] Gao, Q., Tang, W., Yang, Y. & Fu, E. Children’s emotional intelligence and aggressive behavior: the mediating effects of positive affect and negative affect. *Heliyon*10.1016/j.heliyon.2023.e20366 (2023). 37767488 10.1016/j.heliyon.2023.e20366 PMC10520831

[67] Wasik, B. H. Sociometric measures and peer descriptors of kindergarten children: A study of reliability and validity. *J. Clin. Child. Psychol.* **16** (3), 218–224 (1987).

[68] Korkman, M., Kirk, U. & Kemp, S. L. *Administrative Manual* (Psychological Corporation, 2007).

[69] Zelazo, P. D. The dimensional change card sort (DCCS): A method of assessing executive function in children. *Nat. Protoc.* **1**, 297–301. 10.1038/nprot.2006.46 (2006). 17406248 10.1038/nprot.2006.46

[70] Veraksa, A. N., Almazova, O. V. & Bukhalenkova, D. A. Executive functions assessment in senior preschool age: A battery of methods. *Psikhol Zh*. **41**, 108–118. 10.31857/S020595920012593-8 (2020).

[71] Taber, K. S. The use of cronbach’s alpha when developing and reporting research instruments in science education. *Res. Sci. Educ.* **48**, 1273–1296. 10.1007/s11165-016-9602-2 (2018).

[72] Bukhalenkova, D. A. & Almazova, O. V. Experience in applying the CLASS method for assessing the quality of the educational environment of a kindergarten. *Vestnik Moskovskogo Universiteta Ser. 14 Psikhologiya [Moscow Univ. Psychol. Bulletin]*. **2**, 128–152. 10.11621/vsp.2022.02.06 (2022).

[73] Braza, F., et al. Social cognitive predictors of peer acceptance at age 5 and the moderating effects of gender. *Br. J. Dev. Psychol.* **27**(3), 703–716. 10.1348/026151008X360666 (2009). 10.1348/026151008x360666 19994576

[74] Bierman, K. L. & 72 - Bellanti, C. J., & Disentangling the impact of low cognitive ability and inattention on social behavior and peer relationships. *J. Clin. Child. Psychol.* **29**, 66–75. 10.1207/S15374424jccp2901_7 (2000). 10693033 10.1207/S15374424jccp2901_7 PMC2767167

[75] Raven, J., Raven, J. C. & Court, J. H. *Manual for Raven’s Progressive Matrices and Vocabulary Scales. Section 2: The Coloured Progressive Matrices* (Oxford Psychologists, 1998).

[76] Gelman, A. & Hill, J. *Data Analysis Using Regression and Multilevel/hierarchical Models* (Cambridge University Press, 2007).

[77] Kruglanski, A. W. A theory of goal systems. *Adv Exp. Soc. Psychol* **34**. (2002).

[78] Payne, B. K. Conceptualizing control in social cognition: how executive functioning modulates the expression of automatic stereotyping. *J. Pers. Soc. Psychol.* **89** (4), 488 (2005). 16287413 10.1037/0022-3514.89.4.488

[79] Suarez, G. L., Shaw, D. S., Wilson, M. N., Lemery-Chalfant, K. & Hyde, L. W. Inhibitory control in late childhood as a predictor of antisocial behavior in adolescence and the role of social context. *Prev. Sci.* 1–14 (2024). 10.1007/s11121-024-01754-y PMC12086261 39562476

[80] von Hippel, W. & Gonsalkorale, K. That is bloody revolting! Inhibitory control of thoughts better left unsaid. *Psychol. Sci.* **16** (7), 497–500 (2005). 16008778 10.1111/j.0956-7976.2005.01563.x

[81] Marien, H. et al. Being flexible or rigid in goal-directed behavior: when positive affect implicitly motivates the pursuit of goals or means. *J. Exp. Soc. Psychol.* **48**, 277–283 (2011).

[82] Spiegel, J. A., Goodrich, J. M., Morris, B. M., Osborne, C. M. & Lonigan, C. J. Relations between executive functions and academic outcomes in elementary school children: A meta-analysis. *Psychol. Bull.* **147** (4), 329–351. 10.1037/bul0000322 (2021). 34166004 10.1037/bul0000322 PMC8238326

[83] Elkonin, D. B. The psychology of the game. *Pedagogy*. (1978).

[84] Tuzhimeeva, I. I. & Kochisov, V. K. Pedagogical conditions of a personalized approach to teaching in a comprehensive school by means of neuropedagogics. Mir nauki, kul’tury, obrazovaniya [The world of science, culture, education]. **3**(100), 68–71 (2021).

[85] Chief State Sanitary Doctor of the Russian Federation. Decree “On approvalof sanitary rules SP 2.4.3648-20 ‘Sanitary and epidemiological requirements for theorganization of education and training, recreation, and rehabilitation of children andyouth” (September 28, 2020 No. 28). Moscow, Russia. Retrievedfrom https://docs.cntd.ru/document/566085656 (Accessed 10 June 2025) (2020).

[86] Bukowski, W. M., Gauze, C., Hoza, B., & Newcomb, A. F. Differences andconsistency between same-sex and other-sex peer relationships during earlyadolescence.*Dev. Psychol.* **29**(2), 255 (1993).

[87] Williams, B. T. & Gilmour, J. D. *Sociometry and Peer Relationships* (Child Psychology & Psychiatry & Allied Disciplines, 1994). 10.1111/j.1469-7610.1994.tb01806.x 7995849

[88] Coie, J. D. & Dodge, K. A. Continuities and changes in children’s social status: A five-year longitudinal study. *Merrill-Palmer Q* 261–282 (1983).

[89] Guo, L., He, T., Lv, X. & Yu, F. Identifying popular, rejected and neglected children in Chinese preschool: an exploratory study on the educational application of spatial positioning data. *Acta Psychol.* **248**, 104389 (2024). 10.1016/j.actpsy.2024.104389 38970888

[90] Vygotsky, L. S. & Rieber, R. W. (eds). The history of the development of higher mental functions. In *The Collected Works of L. S. Vygotsky*, vol. 4. (ed. Hall, M. J.) (Plenum, 1997).

[91] Nisskaya, A. Social situation of development in a preschool educational institution as a factor of school adaptation in first-graders. *Psychol. Res.* **5** (23), 8. 10.54359/ps.v5i23.777 (2012).

[92] Alonso-Tapia, J. & Nieto, C. Classroom emotional climate: Nature, measurement, effects and implications for education. *Rev. Psicodidact*. **24** (2), 79–87 (2019).

[93] Cordeiro, T., Botelho, J. & Mendonça, C. Relationship between the self-concept of children and their ability to recognize emotions in others. *Front. Psychol.* **12**, 672919. 10.3389/fpsyg.2021.672919 (2021). 34712163 10.3389/fpsyg.2021.672919 PMC8545983

[94] Demirdag, S. Classroom management and students self-esteem: creating positive classrooms. *Educ. Res. Rev.* **10** (2), 191–197 (2015).

[95] Buhs, E. S., Rudasill, K. M., Kalutskaya, I. N. & Griese, E. R. Shyness and engagement: contributions of peer rejection and teacher sensitivity. *ECRQ* **30**, 12–19 (2015).

[96] Hendrickx, M. M., Mainhard, T., Oudman, S., Boor-Klip, H. J. & Brekelmans, M. Teacher behavior and peer liking and disliking: the teacher as a social referent for peer status. *J. Educ. Psychol.* **109** (4), 546 (2017).

[97] Spilles, M., Huber, C., Nicolay, P., König, J. & Hennemann, T. The relationship of classmates-perceived teacher feedback and the social acceptance of second, third and fourth graders. *Int. J. Incl. Educ.* **29** (4), 486–501 (2025).

[98] Wullschleger, A., Garrote, A., Schnepel, S., Jaquiéry, L. & Opitz, E. M. Effects of teacher feedback behavior on social acceptance in inclusive elementary classrooms: exploring social referencing processes in a natural setting. *Contemp. Educ. Psychol.* **60**, 101841 (2020).

[99] Clarke, P. When can group level clustering be ignored? Multilevel models versus single-level models with sparse data. *J. Epidemiol. Community Health*. **62** (8), 752–758 (2008). 18621963 10.1136/jech.2007.060798

[100] Gehlbach, H. et al. Creating birds of similar feathers: leveraging similarity to improve teacher–student relationships and academic achievement. *J. Educ. Psychol.* **108**, 342–352. 10.1037/edu0000042 (2016).

[101] Mikas, D. & Szirovitza, L. School success, gender and sociometric status of students. *Coll. Antropol*. **41** (4), 345–349 (2017).

