# Supplementary material for: Executive functions and classroom quality in kindergarten predict peer acceptance in first grade
Source: Sci Rep. 2025 Dec 4;16:414. doi: 10.1038/s41598-025-30409-3 (PMC12770537; doi:10.1038/s41598-025-30409-3)
Supplement: Supplementary file 1 — Supplementary Material 1 [file 41598_2025_30409_MOESM1_ESM.docx]

**Table S1.** Results of generalized linear models for first graders sociometric status with cluster-robust standard errors

| **Effect** | **β** | **Robust SE** | **C.I. Lower** | **C.I. Upper** | **z** | **p** |
| --- | --- | --- | --- | --- | --- | --- |
| (Intercept) | 0.028 | 0.119 | -0.206 | 0.262 | 0.234 | <.001 |
| Sex | 0.096 | 0.112 | -0.122 | 0.315 | 0.865 | 0.387 |
| Age | 0.028 | 0.052 | -0.074 | 0.129 | 0.533 | 0.594 |
| Non-verbal intelligence | 0.179 | 0.122 | -0.418 | 0.259 | -1.472 | 0.141 |
| Emotional support | 0.190 | 0.134 | -0.453 | 0.373 | -1.419 | 0.156 |
| Classroom organization | 0.073 | 0.123 | -0.315 | 0.168 | -0.595 | 0.552 |
| Instructional support | 0.149 | 0.136 | -0.118 | 0.416 | 1.096 | 0.273 |
| EF (Time 1) | 0.377 | 0.133 | 0.117 | 0.637 | 2.841 | 0.005 |
| Sociometric status (Time 1) | 0.245 | 0.104 | 0.040 | 0.449 | 2.344 | 0.019 |

Note: standardized estimates (β), standard deviation (SD), cluster-robust standard errors (Robust SE).

**Table S2.** Results of generalized linear models for first graders self-sociometric status with cluster-robust standard errors

| **Effect** | **β** | **Robust SE** | **C.I. Lower** | **C.I. Upper** | **z** | **p** |
| --- | --- | --- | --- | --- | --- | --- |
| (Intercept) | 0.117 | 0.164 | -0.206 | 0.439 | 0.709 | <.001 |
| Sex | 0.039 | 0.103 | -0.162 | 0.241 | 0.383 | 0.702 |
| Age | -0.024 | 0.075 | -0.171 | 0.124 | -0.315 | 0.753 |
| Non-verbal intelligence | 0.101 | 0.078 | -0.253 | 0.252 | -1.295 | 0.195 |
| Emotional support | 0.295 | 0.187 | -0.561 | 0.370 | -1.582 | 0.114 |
| Classroom organization | 0.106 | 0.226 | -0.336 | 0.549 | 0.471 | 0.638 |
| Instructional support | 0.240 | 0.168 | -0.089 | 0.569 | 1.431 | 0.152 |
| EF (Time 1) | 0.192 | 0.128 | -0.058 | 0.443 | 1.504 | 0.133 |
| Sociometric status (Time 1) | 0.096 | 0.164 | -0.226 | 0.417 | 0.584 | 0.560 |

Note: standardized estimates (β), standard deviation (SD), cluster-robust standard errors (Robust SE).

**Table S3.** Results of generalized linear models for first graders reciprocity with cluster-robust standard errors

| **Effect** | **β** | **Robust SE** | **C.I. Lower** | **C.I. Upper** | **z** | **p** |
| --- | --- | --- | --- | --- | --- | --- |
| (Intercept) | 0.227 | 0.206 | -0.177 | 0.631 | 1.102 | <.001 |
| Sex | -0.002 | 0.128 | -0.252 | 0.249 | -0.012 | 0.990 |
| Age | 0.017 | 0.038 | -0.057 | 0.091 | 0.451 | 0.652 |
| Non-verbal intelligence | 0.022 | 0.106 | -0.231 | 0.186 | -0.209 | 0.835 |
| Emotional support | 0.127 | 0.167 | -0.454 | 0.201 | -0.759 | 0.448 |
| Classroom organization | 0.112 | 0.181 | -0.467 | 0.242 | -0.621 | 0.534 |
| Instructional support | 0.102 | 0.219 | -0.327 | 0.531 | 0.464 | 0.642 |
| EF (Time 1) | 0.390 | 0.092 | 0.209 | 0.571 | 4.216 | 0.001 |
| Sociometric status (Time 1) | 0.063 | 0.146 | -0.223 | 0.348 | 0.430 | 0.667 |

Note: standardized estimates (β), standard deviation (SD), cluster-robust standard errors (Robust SE).
